# Mercury Spill Responses — Five States, 2012–2015

**DOI:** 10.15585/mmwr.mm6610a3

**Published:** 2017-03-17

**Authors:** Ryan J. Wozniak, Anne E. Hirsch, Christina R. Bush, Stuart Schmitz, Jeff Wenzel

**Affiliations:** ^1^Wisconsin Department of Health Services; ^2^North Carolina Department of Health and Human Services; ^3^Michigan Department of Health and Human Services; ^4^Iowa Department of Public Health; ^5^Missouri Department of Health and Senior Services.

Despite measures to educate the public about the dangers of elemental mercury, spills continue to occur in homes, schools, health care facilities, and other settings, endangering the public’s health and requiring costly cleanup. Mercury is most efficiently absorbed by the lungs, and exposure to high levels of mercury vapor after a release can cause cough, sore throat, shortness of breath, nausea, vomiting, diarrhea, headaches, and visual disturbances (*1*). Children and fetuses are most susceptible to the adverse effects of mercury vapor exposure. Because their organ systems are still developing, children have increased respiratory rates, and they are closer to the ground where mercury vapors are most highly concentrated (*2*). To summarize key features of recent mercury spills and lessons learned, five state health departments involved in the cleanup (Iowa, Michigan, Missouri, North Carolina, and Wisconsin) compiled data from various sources on nonthermometer mercury spills from 2012 to 2015. The most common sites of contamination were residences, schools and school buses, health care facilities, and commercial and industrial facilities. Children aged <18 years were present in about one third of the spills, with approximately one in seven incidents resulting in symptoms consistent with acute mercury exposure. To protect the public’s health after a mercury spill, it is important that local, state, and federal agencies communicate and coordinate effectively to ensure a quick response, and to minimize the spread of contamination. To reduce the number of mercury spills that occur in the United States, public health officials should increase awareness about exchange programs for mercury-containing items and educate school and health care workers about sources of mercury and how to dispose of them properly.

State and local health departments routinely evaluate the cleanup of homes and schools where mercury spills have occurred to ensure that mercury vapor concentrations are reduced to safe levels. Cleanup of elemental mercury is challenging because it is dense and breaks into tiny beads when spilled. Elemental mercury also adheres to surfaces such as shoes, which can promote the spread of contamination, further complicating collection and removal. The Agency for Toxic Substances and Disease Registry (ATSDR) has developed recommended mercury vapor action levels or ranges for different settings to assist health departments with reoccupancy decisions. For residential settings, the ATSDR mercury vapor action level is 1 *µ*g/m^3^; however, concentrations of 1–3 *µ*g/m^3^ are considered acceptable for schools because of the reduced exposure duration (*3*). During 2012–2015, questions related to cleanup of elemental mercury remained the most common type of environmental inquiry received by U.S. poison centers, accounting for 17,498 encounters (including 5,786 for mercury thermometers) and 23% of all environmental inquiries (*4*–*7*). During this period, 11,777 encounters involved elemental mercury exposures, with approximately 93% resulting from unintentional releases and 28% occurring in children aged ≤12 years (*4*–*7*).

After reports that several state health departments responded to significant mercury spills, in March 2015, the Council of State and Territorial Epidemiologists (CSTE) convened a workgroup to compile mercury spill data to increase awareness of the frequency and hazards of mercury spills. Staff members from five state health departments (Iowa, Michigan, Missouri, North Carolina, and Wisconsin) participated in the workgroup and compiled nonthermometer mercury spill data during 2012–2015 from various sources, including internal records, state agencies of emergency management, environmental quality and natural resources, the ATSDR National Toxic Substance Incidents Program, and U.S. Environmental Protection Agency (EPA) on-scene coordinators. Frequency analyses were conducted to summarize key features of the spills, including location and amount, and whether the spill resulted in an official evacuation, children were present, or the spill resulted in symptoms consistent with acute mercury exposure (either medically documented or self-reported). Case studies were collected from each state.

Five state health departments were involved in the cleanup of 64 nonthermometer mercury spills during 2012–2015 (Table 1). The most common sites of contamination were residences (44%), schools and school buses (20%), health care facilities (17%) and commercial and industrial facilities (17%). Approximately 42% of these mercury spills were estimated to involve <0.5 pound (i.e., 1 tablespoon) of mercury, 33% involved >0.5 pound, and 25% involved an unknown amount. A quarter of the mercury spills resulted in an official evacuation, and children aged <18 years were present in at least 35% of the events. Fourteen percent of mercury spills resulted in symptoms consistent with acute mercury exposure, including cough, sore throat, shortness of breath, nausea, vomiting, diarrhea, headaches, and visual disturbances.

**TABLE 1:** Summary of mercury spill data from five state health departments,* 2012—2015

Characteristic	No. (%) of spills
**Total spills**	64 (100)
**Location of spill** ^†^	
Residence	28 (44)
School/School bus	13 (20)
Health care facility	11 (17)
Other commercial/ industrial facility	11 (17)
Water treatment plant	3 (5)
Rest area/ Parking lot/ Street	3 (5)
Penitentiary	1 (2)
**Amount spilled (pounds)**	
<0.5	27 (42)
0.5–1	9 (14)
>1–5	8 (13)
>5	4 (6)
Unknown	16 (25)
**Official evacuation**	
Yes	16 (25)
No	48 (75)
**Potentially exposed children aged <18 years**	
0	32 (50)
1–5	17 (27)
>5	5 (8)
Unknown	10 (16)
**Potentially exposed adults aged ≥18 years**	
0	17 (27)
1–5	26 (41)
>5	11 (17)
Unknown	10 (16)
**Persons with acute symptoms**	
0	54 (84)
1–5	9 (14)
Unknown	1 (2)

* Iowa, Michigan, Missouri, North Carolina, and Wisconsin.

^†^ Some incidents involved multiple locations.

Five cases that occurred during 2012–2014 illustrate the variety of mercury spills to which state health departments were asked to respond.

**Armstrong, Iowa (2012).** A person carried a jar containing approximately 12 pounds of mercury into a bar, where it accidentally spilled. Extensive mercury contamination was found in the bar and in the home of one of the bar patrons. Cleanup in the bar required removing the tile floor, sealing the subfloor, and superheating the indoor air with forced ventilation. Remediation of the home involved extensive cleaning and removal of contaminated items as hazardous waste, including a vacuum cleaner, washer, and clothes dryer. After cleanup of these locations by EPA contractors, mercury vapor monitoring was conducted under typical conditions to confirm that both locations were safe for re-entry. Although the cleanup took one week to complete, no adverse health effects were reported because quick action by responders limited mercury vapor exposure.

**Lenoir, North Carolina (2012).** A student brought a test tube containing mercury to an elementary school. The test tube was dropped in a classroom, spilling approximately 0.5 pounds of mercury. Five exposed students (aged 10–12 years) were taken to a hospital, decontaminated, and released. Multiple federal, state, and local agencies were involved in the response and assessment. Cleanup operations and environmental monitoring were conducted by an environmental contractor and EPA. The school was closed for 2 days before it was cleared for reoccupancy.

**Kansas City, Missouri (2013).** A resident hired a professional clock company to move his antique grandfather clock up a set of stairs. The clock had an estimated 15 pounds of mercury contained in the pendulum. During the move, nearly 2 pounds of mercury were spilled throughout the apartment building. Cleanup of this spill took approximately 2 weeks and resulted in the disposal of the pendulum and the mercury remaining inside it. No adverse health effects were reported among those living at the home.

**Delton, Michigan (2014).** A man attempted to extract gold from jewelry by combining it with elemental mercury and heating the mixture. He was severely poisoned from inhaling very high concentrations of mercury vapors. Multiple federal, state, and local agencies were involved in the cleanup of the home and the medical care of the patient, who survived, but required extensive medical treatment. The home was eventually demolished.

**Bloomer, Wisconsin (2014).** An old mercury-containing boiler was being removed from a home and approximately 3.5 pounds of mercury were released in the basement, garage, and driveway. The state health department provided cleanup guidance and a mercury vapor monitor to assist on-site agencies in overseeing cleanup of this large spill. Professional cleanup of the basement, garage, and driveway required the use of powdered sulfur and a specialized mercury vacuum. The washer and clothes dryer were also contaminated and were discarded as hazardous waste. No adverse health effects were reported by persons living in the home.

## Discussion

As health officials began to understand and appreciate the adverse health effects that exposure to mercury can cause in humans, state and federal agencies began to institute laws and regulations to reduce and control the use, release, and disposal of elemental mercury (*8*).* These regulations have been effective at reducing environmental contamination from industrial and commercial sources; however, numerous stores of elemental mercury still exist in smaller quantities in residences, schools, and health care facilities. Mercury spills can be expensive to clean up to levels considered safe for long-term occupancy, with the cost varying based on the location and extent of contamination. During 2012–2015, EPA reported responding to 225 chemical-release incidents in which mercury was listed as the primary contaminant of concern; the average cost of cleanup to those incidents ranged from approximately $30,000 to $75,000 for each year from 2012 to 2015, and the highest cleanup cost during this time period was $913,915 in 2013 (EPA, unpublished data, December 2015).

Government agencies, academic institutions, and health care and environmental organizations have developed numerous strategies to educate the public about the hazards of elemental mercury and encourage the safe disposal of mercury-containing products (Table 2). For example, ATSDR developed a web-based mercury spill prevention initiative for schools, called “Don’t Mess with Mercury” (http://www.atsdr.cdc.gov/dontmesswithmercury/index.html). The website targets middle schools, providing videos, a game, and lesson plans. EPA has also developed fact sheets with best practices for the removal of mercury-containing devices from residential buildings and health care facilities (*9*,*10*). Despite these efforts, however, mercury spills in these settings continue to occur, requiring costly cleanup to prevent exposures to harmful levels of mercury vapor.

**TABLE 2:** Common sources of mercury in homes, schools and health care facilities — United States, 2001–2017

**Source**	**Approximate amount**	
	**(pounds)**	**(grams)**
Compact fluorescent lightbulbs*	0.00001	0.004
Thermostats (tilt switches)^†^	0.0001–0.0100	0.05–5
Thermometers^§^	0.001–0.020	0.5–10
Float switches^†^	0.0002–0.1500	0.1–70
Blood pressure monitors^§^	0.15–0.20	70–90
Manometers^¶,^**	0.07–0.75	30–340
Gas pressure regulators (residential) ^††^	≤0.3	≤140
Esophageal dilators^§^	≤1.0	≤450
Barometers^§^	≤1.8	≤800
Boiler heating systems^††^	≤3.5	≤1600
Grandfather clocks (pendulum) ^§§^	≤15.0	≤6800

* https://www.epa.gov/cfl/what-are-connections-between-mercury-and-cfls.

^†^ http://www.newmoa.org/prevention/mercury/imerc/factsheets/switches_relays_2014.pdf.

^§^ https://www3.epa.gov/region9/waste/p2/projects/hospital/mercury.pdf.

^¶^ http://www.newmoa.org/prevention/mercury/imerc/factsheets/measuring_devices.cfm.

** http://www.epa.ohio.gov/portals/41/p2/mercury_pbt/manometer_web.pdf.

^††^ https://www.epa.gov/sites/production/files/2015-10/documents/before_you_tear_it_down.pdf.

^§§^ https://www.cdc.gov/mmwr/preview/mmwrhtml/mm5623a2.htm.

Many states have enacted laws against the use of mercury in schools and health care facilities, and instituted bans and phaseouts for the sale of mercury-containing products.* However, many mercury-containing items remain in schools, health care facilities, and homes. Incentivizing persons to relinquish mercury and mercury-containing items through exchange programs (e.g., mercury thermometers for digital thermometers) has been successful in reducing the potential for residential mercury spills, but more awareness of these programs is needed. Although health care workers are aware of the hazards of elemental mercury exposure, they might not be aware of potential mercury sources in health care facilities. Educational programs at schools and hospital grand rounds could help inform school and health care workers about these potential mercury sources and how to dispose of them properly. Although there might be a cost associated with disposing of mercury-containing items properly, that cost is typically far less than the costs incurred to clean up a spill.

The findings in this report are subject to at least three limitations. First, only five states contributed data for this analysis, so the characteristics of mercury spills described in this report might not be representative of all mercury spills that occur in the United States. Second, these data were compiled from many different data sources, so the level of detail available about the spills varied considerably. Third, because the role of state health departments in mercury spill response varies by state, some state health departments responded to mercury spills more frequently than others, and not all mercury spills that occurred are captured in this report.

When mercury spills do occur, a quick and coordinated response is necessary to ensure the protection of public health and proper remediation. When a spill occurs, health departments, local or regional hazardous materials responders, state health and environmental agencies, regional EPA offices, poison control centers, and health care providers should be immediately informed.

**Summary**

**What is already known about this topic?**

Exposure to elemental mercury vapor can cause adverse health effects, especially in children and fetuses. Government agencies and other organizations have tried numerous ways to educate the public about the hazards of elemental mercury and encourage the safe disposal of mercury-containing products.

**What is added by this report?**

Despite measures to educate the public on the dangers of mercury, mercury spills continue to occur in homes, schools, health care facilities, and other settings, requiring costly cleanup to prevent human exposures to harmful levels of mercury vapor. State and local health departments routinely guide the cleanup of buildings where mercury spills have occurred to ensure that mercury vapor concentrations are reduced to safe levels. Illustrative cases of nonthermometer mercury spills in five states are presented, which highlight the extensive use of resources required for remediation, as well as the potential for severe adverse health effects.

**What are the implications for public health practice?**

To protect the public’s health after a mercury spill, it is important that local, state, and federal agencies communicate and coordinate effectively to ensure a rapid response and minimize the spread of contamination. Increasing awareness of exchange programs for mercury-containing items and education of school and health care workers about appropriate disposal might reduce the number of mercury spills that occur in the United States.
